# Upper Limb Deep Venous Thrombosis Associated With Peripherally Inserted Central Catheters in Hospitalised Surgical Patients

**DOI:** 10.7759/cureus.75325

**Published:** 2024-12-08

**Authors:** Nick Li, Paul Njoku, Amit K J Mandal, Jihène El Kafsi, Tom Hosack, Thomas Stockdale, Sohani Dassanayake, Koenraad Van den Abbeele, Jane Democratis, Constantinos G Missouris

**Affiliations:** 1 Surgery, The Queen’s College, University of Oxford, Oxford, GBR; 2 Cardiology, Wexham Park Hospital, Frimley Health NHS Foundation Trust, Slough, GBR; 3 Internal Medicine/Acute Care/Cardiology, Wexham Park Hospital, Frimley Health NHS Foundation Trust, Slough, GBR; 4 Surgery, Ashford and St Peter’s Hospitals NHS Foundation Trust, Chertsey, GBR; 5 Surgery, Wexham Park Hospital, Frimley Health NHS Foundation Trust, Slough, GBR; 6 Medicine, Wexham Park Hospital, Frimley Health NHS Foundation Trust, Slough, GBR; 7 Internal Medicine, Wexham Park Hospital, Frimley Health NHS Foundation Trust, Slough, GBR; 8 Microbiology, Wexham Park Hospital, Frimley Health NHS Foundation Trust, Slough, GBR; 9 Cardiology, University of Nicosia Medical School, Nicosia, CYP

**Keywords:** deep vein thrombosis, general surgery, picc lines, surgical, uldvt, upper limb dvt

## Abstract

Introduction

Surgical inpatients frequently require peripherally inserted central catheters (PICCs) for parenteral feeding and administration of medication. PICCs may cause upper limb deep venous thrombosis (ULDVT), which impacts patient morbidity. We investigated the risk and prevention of PICC-ULDVT in hospitalised surgical inpatients.

Methods

We conducted a retrospective analysis of 168 consecutive surgical inpatients who received a PICC over the same four-year period. Data from 136 additional consecutive general medical inpatients with PICCs over the same period were included for comparison. We observed and compared the incidence of ULDVT confirmed on dedicated upper limb venous imaging in the cohort of surgical patients compared to a time matched cohort of medical patients. We extracted data including patient demographics, details of admission, and insertion site of the PICC from the hospital electronic notes.

Results

The incidence of ULDVT in surgical patients was 4.7% compared to 1.5% in medical patients despite increased age (p=0.001) and comorbidity burden (p=0.001) in the latter group. Ninety percent of surgical patients had abdominal surgery within 30 days of PICC placement. Regression multivariate analysis identified concurrent cancer (p=0.048), median Charlson Comorbidity Index (CCI) (p=0.034), admission with malignant bowel obstruction (p=0.002), and catheter insertion into the brachial vein (p=0.033) as significant risk factors for PICC-ULDVT in hospitalised surgical patients. Standard pharmacological venous thromboembolic events (VTE) prophylaxis, as per national guidelines, did not reduce the risk of PICC-ULDVT.

Conclusion

Our study indicates that surgical inpatients are at greater risk of developing PICC-associated upper limb deep vein thrombosis (ULDVT) compared to medical inpatients, with limited evidence supporting the effectiveness of pharmacological thromboprophylaxis in reducing this risk. PICCs should be used with caution in patients with cancer, cancer-related bowel obstruction, and surgical patients with a higher comorbidity index. PICCs inserted via the basilic vein may reduce ULDVT risk, but further studies are needed on the preferential site of insertion in surgical patients.

## Introduction

Upper limb deep venous thrombosis (ULDVT) comprises 3% of all venous thromboembolic events (VTE) and can cause significant symptoms including swelling, neuropraxia, reduced upper limb function, and embolism to the pulmonary vasculature [1]. Data from the GARFIELD-VTE registry has revealed that invasive central catheters are the sole provoking factor for up to 12% of ULDVT across all patient groups [2]. The contribution of central catheters to inpatient ULDVTs is probably much higher given the increase in overall risk factors such as immobility and the pro-thrombotic state of systemic illness.

Recent surgery has been consistently highlighted as a risk factor for catheter-associated venous thromboembolism [3,4]. Peripherally inserted central catheters (PICCs) are a reliable, safe, and cost-effective method for obtaining central venous access and are increasingly used in surgical patients for parenteral nutrition and medication. However, the risk of PICC-associated ULDVT for hospitalised general surgical patients has not been extensively studied. This patient group is at a higher risk of developing VTE when compared to medical patients, even accounting for surgical inpatients who do not undergo any operative intervention [5-7]. Crucially, pharmacological prophylaxis does not appear effective in preventing catheter-associated ULDVT in ambulating patients, and it remains to be seen if it is effective for the innately high-risk surgical inpatient population [8]. Minimising the incidence of ULDVT in surgical patients being considered for PICCs must focus on recognising and, where appropriate, mitigating risk factors, or otherwise considering alternative options.

In our comparative study, we firstly set out to document the incidence of PICC-associated ULDVT in surgical inpatients compared to medical inpatients hospitalised over a four-year period, secondly to establish risk factors for PICC-associated ULDVT in surgical inpatients, and thirdly to explore whether standard pharmacological VTE prophylaxis is effective in preventing PICC-associated ULDVT for surgical inpatients.

## Materials and methods

Retrospectively, we collected data from electronic records on consecutive general surgical patients in whom a PICC was inserted from 2016 to 2020 at our institution, a large district general hospital in the United Kingdom. The list of patients was provided by the vascular access team who insert the PICCs at our hospital. This allowed for a median follow-up of two years. General surgery patients who were in a critical care setting at the time of PICC insertion were excluded since the use of central venous catheters (CVCs) in critical care patients has been extensively studied. Also, patients within subspecialties such as orthopaedics, vascular surgery, otolaryngology, and urology were excluded as were outpatients and ambulatory clinic patients. A minority of PICCs had been inserted by interventional radiologists; these were also excluded from the analysis. All cases involved PICCs that were inserted by our experienced vascular access practitioners at our institution. Patients with a history of coagulopathies or haematological disorders were also excluded. All PICC lines were inserted using the ultrasound-guided micropuncture technique into brachial or basilic veins. Correct placement of catheter tips in the cavoatrial junction was verified by intracavitary ECG and radiological imaging in all cases. PICC lines were either single, double, or triple lumen and between 4 and 5 French (Fr) in lumen diameter.

A cohort of consecutive acute general medical inpatients and those in associated subspecialties, admitted over the same four-year period with PICCs inserted, were also included for comparison. This gave similar sample sizes for both cohorts. Again, patients with coagulopathies and haematological disorders or in critical or intensive care units and ambulatory or outpatient settings were excluded.

Patient data included age, sex, body mass index (BMI), medical comorbidities (amalgamated using the Charlson Comorbidity Index (CCI), [9] admission diagnosis, recent surgery, cancer diagnosis, indication for the PICC, and the presence of ULDVT or PE. As defined in the literature, a PICC-associated ULDVT was diagnosed by the presence of a ULDVT on imaging, whilst a PICC was in situ in the ipsilateral limb or up to three months after its removal. Gold standard imaging was duplex ultrasonography of the affected limb for ULDVT and computed tomography pulmonary angiography (CTPA) for PE. Data of interest about the PICC included its physical characteristics (diameter, number of lumina, length, and site of insertion), and the duration for which it remained in situ.

Statistical analyses

All statistical analyses were carried out using SPSS (version 29.0, IBM, Chicago, Illinois, USA). For continuous outcome variables, each dataset was tested for normality using Shapiro-Wilk and for significance with either an unpaired t-test if parametric or a Mann-Whitney test if non-parametric. Where possible, outcome sets were normalised by logarithmic transformation and tested for significance using a parametric method. For categorical variables, an odds ratio was calculated and tested for significance using a Pearson Chi-squared test. P-value of <0.05 (two-tailed test) was considered statistically significant.

Ethics

This survey was approved by our institutions’ Research, Quality Improvement and Audit Department with reference FH581, and with clinically collected, non-identifiable data, it does not fall under the remit of NHS Research Ethics Committee. All data was collected locally and handled in accordance with European General Data Protection Regulation (GDPR) standards, as well as local and NHS standards on data protection.

## Results

A total of 168 surgical patients had PICCs inserted by the vascular access team between January 2016 and April 2020. Of these, eight patients (4.8%) were diagnosed with a symptomatic ULDVT. Another three patients (1.8%) developed pulmonary embolism (PE) without evidence of ULDVT or lower limb DVT, and these patients were excluded from subsequent statistical analysis. The baseline demographics of the sample are summarised in Table 1. Differences in gender, age, and CCI between patients who had ULDVT did not reach statistical significance.

**Table 1:** Baseline characteristics of patients admitted under general surgery who required PICC insertion Baseline characteristics of patients admitted under general surgery who required peripherally inserted central catheter (PICC) insertion including gender, age, amalgamated medical comorbidities, and diagnosis necessitating a PICC. PE: pulmonary embolism, ULDVT: upper limb deep venous thrombosis, CCI: Charlson Comorbidity Index.

	ULDVT	PE	No ULDVT or PE
All (n=168)	8	3	157
Male, n (%)	4 (2.4)	0 (0)	91 (54.2)
Female, n (%)	4 (2.4)	3 (1.8)	66 (39.3)
Median age (IQR)	71 (19.75)	70 (17)	63 (22)
Median CCI (IQR)	7.5 (6.25)	5 (2.5)	3 (4)
Diagnosis			
Bowel obstruction, benign	41		
Paralytic Ileus	35		
Bowel obstruction, malignant	24		
Pancreatitis	21		
Inflammatory bowel disease	19		
Bowel perforation	14		
Trauma	2		
Other	12		

Pharmacological prophylaxis and ULDVT

Out of 165 surgical inpatients, 156 (94.5%) received pharmacological VTE prophylaxis according to the National Institute for Health and Care Excellence (NICE) guidelines [10] or were taking pre-existing oral anticoagulation for the duration of their admission. Eight (4.8%) patients were diagnosed with PICC-associated ULDVT, of which seven (87.5%) received pharmacological VTE prophylaxis from admission to the time of diagnosis. A total of 157 patients did not develop ULDVT, of which 149 received pharmacological prophylaxis. The relative risk for developing ULDVT in the group receiving VTE prophylaxis compared to the group who did not was 0.403 (95% CI: 0.139-7.369). Furthermore, in univariate, multivariate, and Chi-squared tests, pharmacological VTE prophylaxis did not significantly affect the risk of PICC-associated VTE (p-values=0.38, 0.52 and 0.49, respectively).

Risk factors for PICC-associated ULDVT

**Figure 1:**
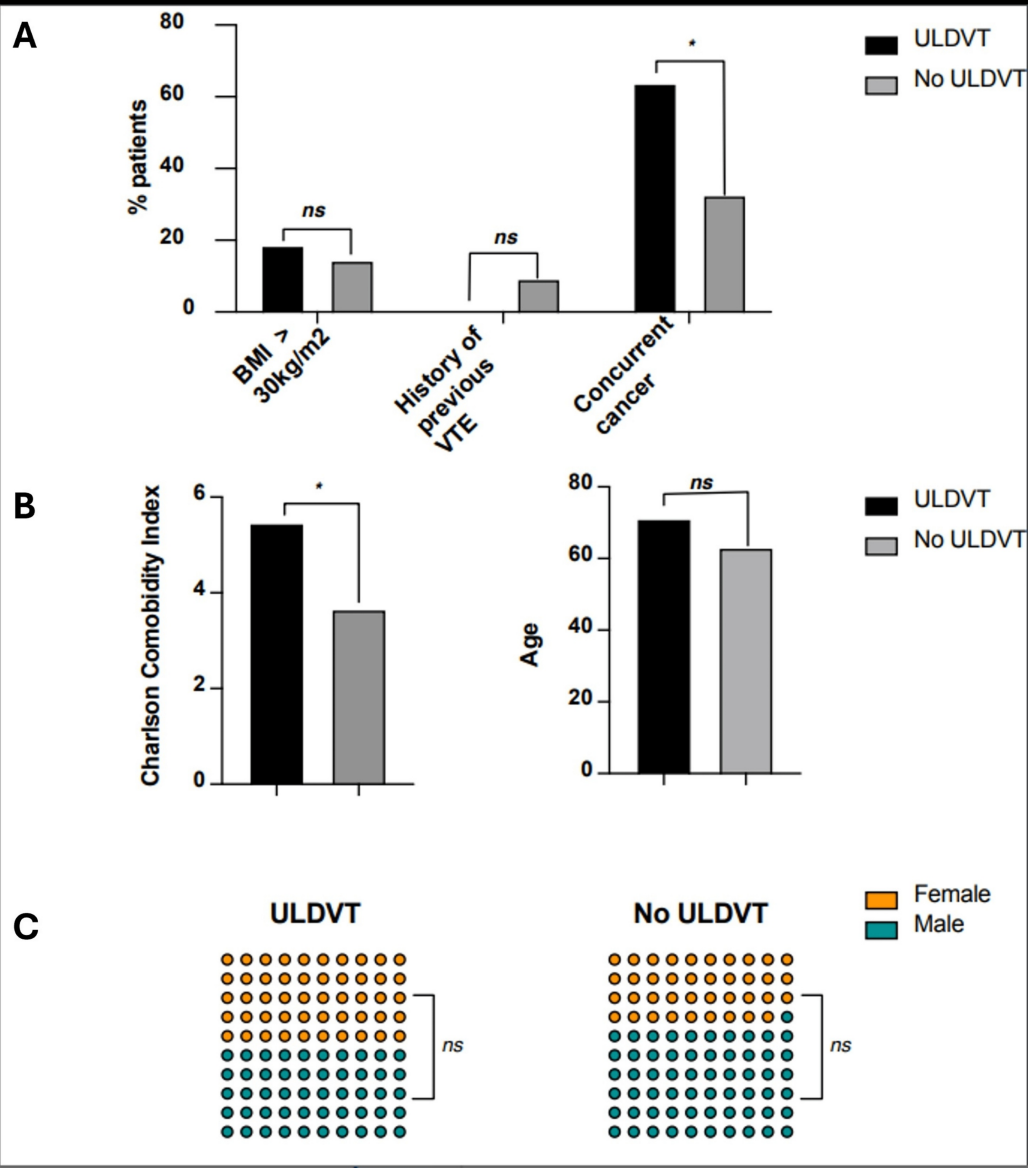
Patient-related risk factors for PICC-associated ULDVT in surgical inpatients Patient-related risk factors for peripherally inserted central catheter (PICC)-associated upper limb deep vein thrombosis (ULDVT) in surgical patients. * denotes statistical significance at 95% confidence interval. ’ns’ denotes no statistical significance at the 95% confidence interval. Statistical analysis was performed using the Mann-Whitney U-test for continuous variables; median age and Charlson Comorbidity Index (CCI), and Chi-squared test for categorical variables; gender, BMI > 30 kg/m^2^, history of previous VTE. A: Barchart displaying incidence of ULDVT in groups with BMI > 30 kg/m^2^, history of venous thromboembolism (VTE), and concurrent cancer. B: Barchart comparing the median Charlson Comorbidity Index (left barchart) and median age (right barchart) in groups of patients who had ULDVT compared to cases without ULDVT. C: Dot Matrix Chart displaying the distribution of females to males in the cohort of patients with ULDVT (left image) and without ULDVT (right image).

Risk factors for ULDVT were categorised into three groups: factors relating to the patient demographics and medical history, factors relating to the disease which necessitated admission, and physical characteristics of the PICC lines. Patient factors are summarised in Table 2 and Figures 1A-1C. These include age, gender, obesity (defined as BMI >30), history of previous VTE, and the CCI as a measure of medical comorbidity. The presence of active cancer or cancer treatment on admission was also included, irrespective of stage or primary site. The CCI and the presence of cancer were significantly associated with the development of ULVT (p= 0.034 and 0.048, respectively) (Table 2).

**Table 2:** Patient-related risk factors for PICC-ULDVT in surgical inpatients Patient-related risk factors for peripherally inserted central catheters (PICC)-associated upper limb deep vein thrombosis (ULDVT) in surgical inpatients. *denotes statistical significance at the 95% confidence interval with multivariate analysis. A p-value of less than 0.05 was considered statistically significant.

Patient factors	ULDVT	No ULDVT	p-value
Median age (yrs)	71	63	0.323
Gender (% female)	50	42.7	0.108
BMI (% of patients >30 kg/m^2^)	18.2	14.3	0.273
History of previous VTE (% yes)	0	9.18	0.301
CCI (median)	5.45	3.65	0.034*
Concurrent cancer (%yes)	63.6	32.48	0.048*

Factors relating to the admission included whether emergency or elective, diagnosis, and whether the patient underwent major surgery within 30 days of PICC placement. For patients with an admission diagnosis of cancer-related bowel obstruction, there was a significant association with the development of ULDVT (p=0.002) (Table 3). Multivariate analysis accounted for the concurrence of cancer and obstruction. Major surgery was performed in over 90% of patients with PICC-associated ULDVT and 67% without ULDVT. However, this did not reach statistical significance with multivariate analysis.

**Table 3:** Disease-related factors for PICC-ULDVT in surgical inpatients Disease-related factors for peripherally inserted central catheters (PICCs)-associated upper limb deep vein thrombosis (ULDVT) in surgical inpatients. * denotes statistical significance at the 95% confidence interval with multivariate analysis. A p-value of less than 0.05 was considered statistically significant.

Disease factors	ULDVT	No ULDVT	p-value
Elective or emergency (% emergency)	90.9	82.2	0.461
Surgery within 30 days of PICC insertion (% yes)	90.1	67.6	0.106
Median duration of PICC in situ (days)	11.5	12.0	0.891
Diagnoses			
Benign bowel obstruction	3	38	0.820
Malignant bowel obstruction	5	19	0.002*
Pancreatitis and associated complications	1	20	0.726
Inflammatory bowel disease	0	19	0.223
Ileus	1	34	0.324
Perforation	0	14	0.304
Trauma	0	2	0.709
Other	1	11	0.797

Duration of PICC in situ and ULDVT

The median duration for which PICCs remained in situ was 11.5 days in patients who developed a ULDVT and 12 days in patients who did not. There was no significant difference between the two groups (p=0.891) (Table 3). Six patients developed ULDVT within a week following PICC insertion. In four of the eight patients with PICC-associated ULDVT, the PICC was removed within 24 hrs of diagnosis. The remaining two developed ULDVT over 50 days after PICC insertion and over 30 days after removal of the PICC.

Physical characteristics of the PICCs were recorded (Table 4). PICC lines contained between one to three lumina. The diameter of each lumen was either 4- or 5-French catheter scale (Fr). The PICCs were inserted into the basilic or the brachial vein. The total length of the PICC in situ and the site of insertion was also recorded. Insertion into the brachial vein compared to the basilic vein was significantly associated with the development of ULDVT (p=0.033).

**Table 4:** Physical characteristics of PICC lines and their association with ULDVT in surgical inpatients Physical characteristics of peripherally inserted central catheters (PICCs) lines and their association with upper limb deep vein thrombosis (ULDVT) in surgical inpatients. * denotes statistical significance at the 95% confidence interval with multivariate analysis. A p-value of less than 0.05 was considered statistically significant.

PICC characteristics	ULDVT	No ULDVT	p-value
Median no. of lumina	2	2	0.602
Median diameter (Fr)	5	5	0.177
Side of insertion (% Left)	36.5	21.1	0.227
Site of insertion (% Brachial vein)	87.5	71.3	0.033*
Median length inserted (cm)	44.5	43	0.990

Comparison with medical patients

A time-matched dataset was collected for 132 patients admitted under general medicine for comparison. A total of 125 (95%) medical inpatients received pharmacological VTE prophylaxis or were on oral anticoagulant therapy started pre-admission. The prevalence of ULDVT was significantly lower in the medical cohort (1.5% compared to 4.7%; p=0.034), despite a significantly higher age and CCI, both of which were associated with ULDVT in surgical patients (Table 5).

**Table 5:** Comparison of demographics between medical and surgical inpatients with peripherally inserted central catheter-associated upper limb deep vein thrombosis Comparison of demographics between medical and surgical inpatients with peripherally inserted central catheter (PICC)-associated upper limb deep vein thrombosis (ULDVT). * denotes statistical significance at the 95% confidence interval with multivariate analysis. A p-value of less than 0.05 was considered statistically significant.

	Medical inpatients	Surgical inpatients	p-value
Median age (IQR)	74 (24)	63 (23)	0.001*
Male	64	95	0.166
Female	68	73	
PICC-associated ULDVT	2	8	0.034*
Median CCI (IQR)	5 (4)	3 (3.5)	0.001*

## Discussion

Peripherally inserted central catheters (PICCs) are widely used in healthcare settings to establish long-term venous access for the provision of parenteral nutrition, the administration of medications, and for patients who require frequent blood sampling. Indwelling PICC lines are generally safe; however, their use can be complicated by ULDVTs and subsequent embolisation, with the associated risk being dependent on a number of thrombogenic risk factors [11].

Whilst several risk factors are non-modifiable, certain VTE risk factors such as the choice of catheter size and insertion technique are significant [12,13] and should be considered to reduce the risk of PICC-associated ULDVT. Catheter-to-vein ratio (CVR) not exceeding 45% of the cross-sectional area of the cannulated vein has been shown to confer a reduced risk of PICC-related thrombosis [14,15]. The technical factors considered during PICC line insertion have a role to play in the incidence of PICC-associated ULDVT [13]. In this study, all PICC insertions were performed under ultrasound guidance with appropriate catheter size choice in relation to the choice of vein. Verification of tip location was performed using intracavitary ECG and X-ray imaging. As catheter-to-vein ratio (CVR) was standardised (<45%) across the entire patient cohort, CVR was not considered during statistical analysis.

The results of this study clearly demonstrate that having major abdominal surgery or being admitted with an abdominal surgical condition heightens risk of developing ULDVT with PICCs. In our study, the incidence of PICC-associated ULDVT among general surgical inpatients was 4.7% compared to 1.5% in general medical inpatients, despite the higher age and burden of comorbidity in the latter group. Ninety percent of the surgical patients who developed ULDVT had major surgery within 30 days. There was a statistically significant association with concurrent diagnosis of cancer, cancer-related bowel obstruction, higher median CCI, and catheter insertion into the brachial vein. We have also determined that standard pharmacological VTE prophylaxis given to surgical inpatients (according to NICE guidance) is not sufficient to prevent line associated ULDVT. This highlights the need for more research to provide evidence-based guidance aimed at mitigating VTE risk or effectively treating ULDVT in surgical patients.

The largest and most recent meta-analysis (75 articles and 109,292 patients) demonstrated that the incidence of PICC-associated symptomatic VTE (DVT and PE) was 3.7% in hospitalised patients. On subgroup analysis, frequency of VTE was highest in patients who were in critical/intensive care, at 10.6%. A major limitation was the pool of non-comparative studies with significant heterogeneity in terms of design, sample size, measurement indices, and outcomes [6]. General surgical inpatients are at higher risk of developing all VTE and commonly require the use of PICCs as the oral route is often unavailable for nutrition and medication. Our study is the first that we are aware of to focus on PICC-associated ULDVT in a cohort of general surgical inpatients.

Almost all surgical inpatients are given pharmacological VTE prophylaxis on admission, barring those with obvious contraindications such as concomitant use of anticoagulants, or a high bleeding risk such as active bleeding, or clotting disorders. Pharmacological VTE prophylaxis is also usually not held for general surgical procedures as it does not result in higher intra-operative or post-operative bleeding risk [5]. Alongside other studies, our data indicates that standard pharmacological VTE prophylaxis does not prevent PICC-associated ULDVT in surgical inpatients. In a study of ambulating cancer outpatients, pharmacological prophylaxis with direct oral anticoagulants did not decrease the incidence of line-associated venous thrombosis. The authors postulate that this was due to the thrombogenic nature of the foreign material itself or the endothelial disruption caused by line insertion and continued endothelial agitation whilst in situ [7]. As such, PICC-associated ULDVT may be considered a direct consequence of PICC insertion and intimal injury, the risk being increased by other systemic factors. This necessitates further research to evaluate the optimum VTE chemoprophylaxis within the context of PICC insertion, particularly for patients at augmented risk. At the time of writing, anticoagulation with low molecular weight heparin, vitamin K agonists, or direct oral anticoagulants (DOACs) for a minimum of three months is an accepted treatment for PICC-related ULDVT, despite any specific randomised controlled trials. In addition, it is unclear whether the PICC can remain in situ when functional and clinically necessary in the presence of PICC-related DVT [3,16,17].

PICCs appear more thrombogenic than centrally inserted catheters. A possible explanation is the predominant use of central lines placed through the internal jugular vein which is of large calibre and bypasses the venous drainage of the upper limb. The ubiquitous use of centrally inserted catheters in the intensive care setting may partly explain the unexpected lower incidence of VTE within this subset of patients [18]. Another consideration is the increased rate of coagulopathy seen in critically unwell patients. The use of dressings impregnated with chlorhexidine has been shown to reduce the incidence of central line-associated thrombus in critical care patients [19,20]. The mechanism of action for this has not been described in detail but is purportedly due to thrombin inhibition by chlorhexidine. A randomised control trial into whether chlorhexidine-impregnated PICCs can reduce the risk of line-associated thrombosis and infection is currently ongoing [20].

In our multivariate analysis of risk factors for PICC-associated ULDVT, specifically in surgical patients, we identified (i) concurrent cancer, (ii) higher CCI, (iii) admission with malignant bowel obstruction, and (iv) cannulation of the brachial vein as significant risk factors. Also, in this cohort, general VTE risk factors such as BMI, age, and past medical history of VTE did not predispose to the incidence of PICC-associated ULDVT. Whilst undoubtedly multifactorial in aetiology, this supports the view that a major risk factor for catheter-associated thrombus might be the thrombogenic properties of the foreign material itself. Although not specific to hospital inpatients, another cohort study also reported that previous DVT and age are not risk factors for line-associated thrombosis which is consistent with the findings of this study [21].

Our data suggests that the site of insertion of PICCs was an important risk factor in the development of ULDVT. Use of the deep brachial vein was significantly associated with greater risk of ULDVT, compared to insertion directly into the superficial basilic vein. Both vessels are of similar calibre (approximately 5 mm). We postulate that direct endothelial damage through cannulation of the more distal brachial vein, which has less tributaries than the basilic vein, may result in more significant haemodynamic flow changes and stasis, and therefore an increased risk of ULDVT. However, a retrospective study evaluating upper extremity PICC DVT in both medical and surgical inpatients and outpatients reported a lower incidence of PICC-ULDVT in the brachial vein compared to the basilic vein (2.2% and 3.1%, respectively) [22]. Additional research is therefore required to determine the most suitable upper limb vein for PICC insertion.

The retention time of the PICC did not appear a significant risk factor for developing ULDVT. Most patients with PICC-associated ULDVT were diagnosed within a week of PICC insertion, and median retention times did not differ between patients who developed ULDVT and those who did not. Therefore, it appears that the incidence of ULDVT did not increase with duration of PICC retention. This is an important observation as many general surgical patients require long-term parenteral nutrition.

There is wide variation in the literature with respect to the incidence of ULDVT within the context of PICCs, and there is no existing validated clinical risk stratification tool. The Michigan Risk score for PICC-related thrombosis [23] is a risk stratification tool that is yet to be externally validated or validated amongst surgical inpatients. It incorporates risk factors such as previous VTE, leucocytosis, the presence of another CVC, number of PICC lumens, and active cancer to assess the probability of VTE if a PICC was inserted in the medical setting. Our study demonstrates the importance of considering additional risk factors for PICC-associated VTE, namely the site of insertion and comorbidity burden (CCI) when evaluating the risk of VTE in surgical patients.

Limitations

As a single-centre study at a district general hospital, we cannot generalise our results to other settings. Moreover, our trust is based in southern England, where population demographics and socioeconomic status may differ from those elsewhere. Median age and comorbidity burden although similar were statistically significant between the medical and surgical cohorts. However, the effects of potential confounders were controlled using multivariate analysis.

Considerations were made for the utilisation of sample size calculations to ensure the study was powered appropriately. Despite achieving significant results in a number of outcomes, we acknowledge the risk of a type 2 error occurring with our experimental sample size. Therefore, we were unable to discount an association between ULDVT and PICCs and those variables which did not achieve statistical significance with observed effect sizes.

There is evidence linking vein calibre used in PICC insertion or catheter-to-vein ratio (CVR), to the risk of upper limb VTE. Evidence suggests a CVR of ≤45% confers a reduced risk of PICC-related thrombosis [14,15]. CVR or average vein diameter was not reported in this study; however, all PICCs were inserted into appropriately sized veins and CVR was standardised across cohorts. We also acknowledge that extracting information from medical notes requires second-hand interpretation and may not be representative of the full clinical picture. There were also limitations in that we relied on observational, retrospective data.

## Conclusions

Surgery itself is a major risk factor for the development of VTE. The incidence of PICC-related ULDVT in this general surgical cohort was 4.8%, and PICCs should be used with caution in patients with cancer and multiple comorbidities. Standard VTE prophylaxis did not appear to prevent PICC-associated ULDVT. A simple modifiable risk factor is the choice of the basilic vein primarily for PICC insertion. We recommend further comparative studies with larger sample sizes to be conducted to generate validated risk stratification models to identify patients at higher risk for PICC-related ULDVT and to provide guidelines for effective pharmacological thromboprophylaxis.
